# Deep Brain Stimulation Can Differentiate Subregions of the Human Subthalamic Nucleus Area by EEG Biomarkers

**DOI:** 10.3389/fnsys.2021.747681

**Published:** 2021-10-20

**Authors:** Daniel Sand, David Arkadir, Muneer Abu Snineh, Odeya Marmor, Zvi Israel, Hagai Bergman, Sharon Hassin-Baer, Simon Israeli-Korn, Ziv Peremen, Amir B. Geva, Renana Eitan

**Affiliations:** ^1^Department of Medical Neurobiology (Physiology), Institute of Medical Research Israel-Canada, Hebrew University of Jerusalem, Jerusalem, Israel; ^2^Edmond and Lily Safra Center for Brain Research, Hebrew University of Jerusalem, Jerusalem, Israel; ^3^Elminda Ltd., Herzliya, Israel; ^4^Department of Neurology, Hadassah Medical Center and Faculty of Medicine, Hebrew University of Jerusalem, Jerusalem, Israel; ^5^Brain Division, Hadassah Medical Organization and Faculty of Medicine, Hebrew University of Jerusalem, Jerusalem, Israel; ^6^Functional Neurosurgery Unit, Hadassah Medical Organization and Faculty of Medicine, Hebrew University of Jerusalem, Jerusalem, Israel; ^7^Department of Neurology, Movement Disorders Institute, Sheba Medical Center and Sackler School of Medicine, Tel Aviv University, Tel Aviv, Israel; ^8^Department of Electrical and Computer Engineering, Ben Gurion University, Beer-Sheva, Israel; ^9^Neuropsychiatry Unit, Jerusalem Mental Health Center and Faculty of Medicine, Hebrew University of Jerusalem, Jerusalem, Israel; ^10^Department of Psychiatry, Brigham and Women's Hospital, Harvard Medical School, Boston, MA, United States

**Keywords:** deep brain stimulation (DBS), Parkinson's disease, postoperative contact selection, EEG, biomarker, machine learning, subthalamic nucleus (STN), zona incerta

## Abstract

**Introduction:** Precise lead localization is crucial for an optimal clinical outcome of subthalamic nucleus (STN) deep brain stimulation (DBS) treatment in patients with Parkinson's disease (PD). Currently, anatomical measures, as well as invasive intraoperative electrophysiological recordings, are used to locate DBS electrodes. The objective of this study was to find an alternative electrophysiology tool for STN DBS lead localization.

**Methods:** Sixty-one postoperative electrophysiology recording sessions were obtained from 17 DBS-treated patients with PD. An intraoperative physiological method automatically detected STN borders and subregions. Postoperative EEG cortical activity was measured, while STN low frequency stimulation (LFS) was applied to different areas inside and outside the STN. Machine learning models were used to differentiate stimulation locations, based on EEG analysis of engineered features.

**Results:** A machine learning algorithm identified the top 25 evoked response potentials (ERPs), engineered features that can differentiate inside and outside STN stimulation locations as well as within STN stimulation locations. Evoked responses in the medial and ipsilateral fronto-central areas were found to be most significant for predicting the location of STN stimulation. Two-class linear support vector machine (SVM) predicted the inside (dorso-lateral region, DLR, and ventro-medial region, VMR) vs. outside [zona incerta, ZI, STN stimulation classification with an accuracy of 0.98 and 0.82 for ZI vs. VMR and ZI vs. DLR, respectively, and an accuracy of 0.77 for the within STN (DLR vs. VMR)]. Multiclass linear SVM predicted all areas with an accuracy of 0.82 for the outside and within STN stimulation locations (ZI vs. DLR vs. VMR).

**Conclusions:** Electroencephalogram biomarkers can use low-frequency STN stimulation to localize STN DBS electrodes to ZI, DLR, and VMR STN subregions. These models can be used for both intraoperative electrode localization and postoperative stimulation programming sessions, and have a potential to improve STN DBS clinical outcomes.

## Introduction

Subthalamic deep-brain stimulation (DBS) is an effective treatment for advanced Parkinson's disease (PD). The therapeutic effect of DBS correlates with accurate localization of the electrode contact in the STN target (Buhmann et al., 2017; Obeso et al., 2017). There are currently two main methods for DBS electrode localization, which can be used independently or as supplementary methods. One is anatomic localization by pre- or intraoperative MRI imaging (and postoperative CT validation). The other is physiological localization using intraoperative STN microelectrode recordings (MERs).

Most clinical centers worldwide perform 3-Tesla MRI imaging for surgical planning and postoperative CT with registration software for the validation of electrode position. Although imaging technology has improved, imaging quality remains a critical factor in the precise localization of DBS electrodes (Ewert et al., 2018; Husch et al., 2018). Higher resolution 7-Tesla MRI improves accuracy but is unavailable in most clinical settings (Verhagen et al., 2016; Bot et al., 2019). Machine learning methods have been suggested for improving 3-Tesla MRI accuracy, but their clinical usage has not become common (Shamir et al., 2019).

Intraoperative MERs enable the physiological localization of DBS electrodes. Although MER localization is very accurate (0.1-mm resolution), the procedure may increase the risk of bleeding or infection (Kimmelman et al., 2011; Ho et al., 2018). MERs can outline STN borders and define functional areas within the STN, such as the dorso-lateral (motor) region (DLR) and the ventral-medial (limbic-cognitive) region (VMR) (Moran et al., 2006; Zaidel et al., 2010; Rappel et al., 2020). Most recently, advanced tools have been introduced to enable an automatic and faster detection of STN borders (Valsky et al., 2016, 2020; HaGuide, Neuro-OmegaTM). Nevertheless, developing non-invasive tools to improve the invasive DBS procedure, intra- and postoperatively, remains of great importance. The objective of this study was to develop a non-invasive EEG-based physiological tool for localizing invasive DBS electrodes. While the effect of DBS stimulation on cortical activity has been studied, few investigations have demonstrated cortical potentials at 1–400 ms after STN-DBS (Ashby et al., 2001; Baker et al., 2002; Walker et al., 2012; Irwin et al., 2020). Our study used the postoperative EEG cortical activity features (DBS-evoked responses) of 17 STN DBS-treated patients with PD. We quantified, for the first time, cortical activity differences evoked within and without the STN in order to find biomarkers to differentiate between these subregions. We also built machine learning (ML) models that can use EEG cortical activity to identify the exact STN lead location.

## Methods

### Patients

Seventeen STN DBS-treated patients with PD (13 males, four females) were recruited in two medical centers in Israel, the Hadassah Medical Center (12 subjects) and Sheba Medical Center (five subjects) (Table 1). All met the clinical inclusion criteria of the study, and were competent to consent (as measured by Mini-Mental State Examination [(MMSE) scale > 26 Roalf et al., 2013] and signed an informed consent. All EEG recordings were taken at least 3 weeks after DBS surgery. The study was authorized by the local IRB Committee of Hadassah Medical Center [no. 0403-13-HMO, NIH clinical trials registration (no. NCT01590056)] and local IRB committee at Sheba Medical Center (no. 3496-16-SMC, NIH clinical trials registration NCT01590056).

**Table 1:** Clinical stimulation characteristics of the analyzed patients.

**Patient**	**Medical Center**	**Sex**	**Age (years)**	**Clinical settings**	**Stimulus frequency (Hz)**	**Recording time/ contact**	**Total no. of stimuli/ contact**	**EGI recording system**
S1	Hadassah	M	76	130 Hz, 1.4 V, 60 μs. 1- IPG case+	3	13 min 20 s	2,400	EGI 128 channels
S2	Hadassah	M	65	130 Hz, 1.6 V, 60 μs. 1- IPG case+	3	13.3 min	2,400	EGI 128 channels
S3	Hadassah	M	61	170 Hz, 2.4 V, 90 μs. 1- IPG case+	3	13.3 min	2,400	EGI 128 channels
S4	Hadassah	M	56	150 Hz, 2.9 V, 60 μs. 1- IPG case+	3	13.3 min	2,400	EGI 128 channels
S5	Hadassah	F	60	125 Hz, 1.4 V, 60 μs. 1- IPG case+	5	8 min	2,400	EGI 128 channels
S6	Hadassah	F	60	125 Hz, 1.8 V, 60 μs. 1- 0+	5	8 min	2,400	EGI 128 channels
S7	Hadassah	M	78	150 Hz, 3.2 V, 60 μs. 1- IPG case+	5	8 min	2,400	EGI 128 channels
S8	Hadassah	M	55	180 Hz, 2.1 V, 60 μs. 1- IPG case+	5	8 min	2,400	EGI 128 channels
S9	Hadassah	F	74	130 Hz, 0.8 V, 60 μs. 1- IPG case+	5	8 min	2,400	EGI 128 channels
S10	Hadassah	M	57	150 Hz, 3.4 V, 60 μs. 1- IPG case+	5	8 min	2,400	EGI 128 channels
S11	Hadassah	M	65	160 Hz, 2.4 V, 60 μs. 0- 2- IPG case+	5	8 min	2,400	EGI 128 channels
S12	Hadassah	M	67	150 Hz, 2.6 V, 60 μs. 0- IPG case+	5	8 min	2,400	EGI 128 channels
S13	Sheba	M	62	120 Hz, 3.1 V, 60 μs. 1- 2- 3+	5	8 min	2,400	EGI 64 channels
S14	Sheba	M	65	130 Hz, 2.8 V, 60 μs. 3- IPG case+	5	8 min	2,400	EGI 64 channels
S15	Sheba	M	66	130 Hz, 2.2 V, 60 μs. 3- IPG case+	5	8 min	2,400	EGI 64 channels
S16	Sheba	F	72	130 Hz, 1.8 V, 60 μs. 2- IPG case+	5	8 min	2,400	EGI 64 channels
S17	Sheba	M	51	130 Hz, 1 V, 60 μs. 2- IPG case+	5	8 min	2,400	EGI 64 channels

### Intraoperative Procedure

**Figure 1:**
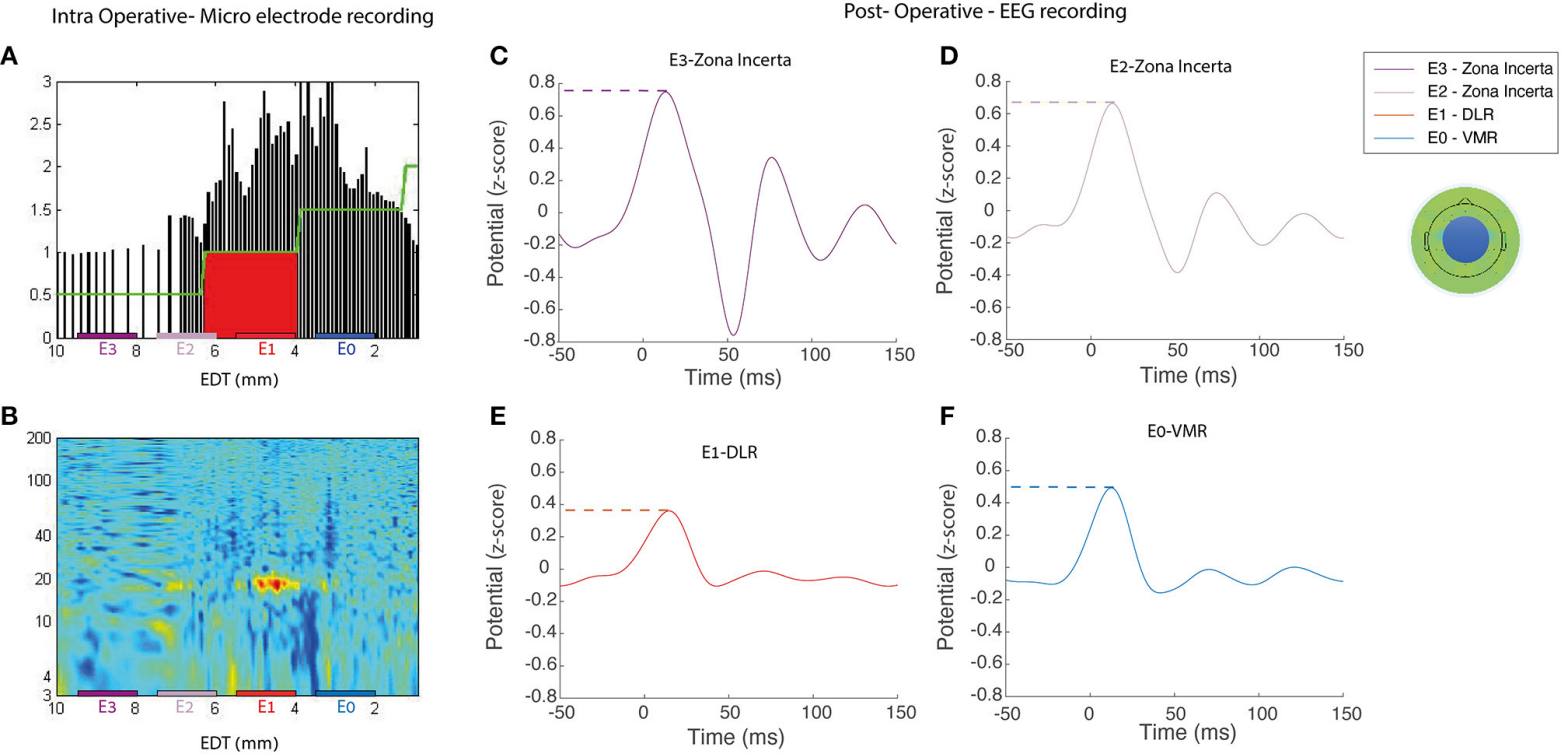
Representative example of intra- and postoperative recordings. An example of intraoperative and postoperative recordings for a single subject (no S8) (left) Microelectrode recording during the DBS implantation in the right STN, the normalized root mean squared [NRMS, **(A)**], power spectral density [PSD, **(B)**] in a contact location of a patient STN. EDT, estimated distance to target (defined as STN center, according to preoperative imaging). **(C–F)** ERP activity of the average EEG electrodes in the medial frontal central area (represented by the blue circle on the topoplot) reveals postoperative stimulations by each of the four DBS contacts; dashed color lines represent the max-peak feature and one of the extracted engineered features. It can also be observed that the signal arises slightly before stimulus onset. Rather than a detection error, this results from the effect of the filter on the interpolated signal, which replaced the stimulus artifact. DLR, dorso-lateral region; VMR, ventro-medial region.

The surgical and recording techniques used in our study have been described elsewhere (Zaidel et al., 2009; Marmor et al., 2017; Rappel et al., 2018; Sand et al., 2021). Briefly, STN target coordinates were chosen using the Framelink 5 or Cranial software (Medtronic Inc., Minneapolis, MN, United States). Neurophysiological data were acquired with the NeuroOmega system (AlphaOmega Engineering, Nazareth, Israel). The analysis of the neurophysiological intraoperative recordings has also been described (Marmor et al., 2017; Rappel et al., 2018; Sand et al., 2021). STN entry and exit were detected automatically with a Hidden Markov Model (HMM) (Zaidel et al., 2009; Valsky et al., 2016). STN trajectories included the DLR and VMR domains of the STN. Each STN recording site was classified as DLR or VMR according to the HMM algorithm and real-time tagging by an experienced electrophysiologist (HB and OM). DLR regions were classified by the beta-oscillatory activity, and VMR regions by its absence. An example of the intraoperative STN recordings, shown in Figures 1A,B, demonstrates the normalized root mean square (RMS) of the spiking activity (300–9,000 Hz) and the power spectral density (PSD). At the end of intraoperative recording and location verification, the two recording electrodes were removed, and the permanent lead was implanted in the preferred trajectory (Activa PC; Medtronic, Inc., Minneapolis, MN, United States). The lead contains four contacts, numbered from E0 (ventral) to E3 (dorsal), with a diameter of 1.27 mm and a length of 1.5 mm, spaced at 0.5 mm intervals.

### Study Procedure

All the patients were asked to stop dopaminergic treatment the night before the study. In all the recording sessions, the patients were seated in a quiet room, awake, and instructed to avoid body movements and focus their gaze at a fixed object at a 70-cm distance. The sessions began with a baseline recording after DBS had been turned OFF for 1 min. All the patients then had a right hemisphere low-frequency stimulation (LFS) for a few minutes (5 Hz for 8 min or 3 Hz for 13.3 mi, in both instances 2,400 stimulations, voltage at 2 V, pulse width at 60 μs), followed by a 1-min OFF DBS stimulation period. Each stimulation session was applied to one of the four DBS contacts. The order of the contact stimulated was determined by a pseudo-random number generator (Haahr, 2009). Most of the patients (*n* = 13) completed the recordings with stimulation of four DBS contacts. A small number (*n* = 4) of patients completed the recordings with one, two, or three DBS contacts (2,1,1, respectively), due to fatigue or discomfort from a prolonged OFF state due to being both OFF medications and OFF clinical DBS. The four contacts of the patients were classified into the three STN subregions (zona incerta, DLR, and VMR) according to the intraoperative recordings. Contacts placed partially in two subregions were classified according to the location of the larger part of the contact. A total of 61 recording sessions were analyzed, with contacts located in the ZI (*n* = 20), DLR (*n* = 23), and VMR (*n* = 18) subregions. At the end of the study procedure, the patients were restored to their baseline dopaminergic treatment and DBS parameters.

### EEG Recordings and Cleaning Process

Electroencephalogram recordings were sampled at 1,000 Hz with HydroCel Geodesic Sensor Netfrom 64 (Electrical Geodesic Inc., Eugene, OR, United States) (five patients) and 128 (12 patients) channels, and a Net Amps 400 amplifier (Electrical Geodesic Inc., Eugene, OR, United States) (Tucker, 1993). Cz was the reference recording electrode. EEG recordings with the 128 channels system were interpolated to 64 channels to enable a unified dataset.

Electroencephalogram signal cleaning was performed as follows. First, the direct current (DC) was subtracted from each electrode. Second, DBS artifacts were automatically detected, based on a threshold of 3^*^ standard deviations (SD) above the raw average (Supplementary Figure 1) and replaced with interpolation using the polynomial curve-fitting of first degree. Third, the EEG signal was referenced to the average reference of all channels. Fourth, the signals were filtered with a high-pass filter of 0.5 Hz and a low-pass filter of 40 Hz, by two-way least-squares finite impulse response (FIR) filtering, implemented in EEGLAB (Delorme and Makeig, 2004).

### STN Evoked Response Potentials and Feature Processing

The STN evoked response potentials (STN ERPs) were acquired by recording cortical EEG signals, while the DBS lead stimulated the STN. In this study, the term “STN ERPs” refers to the EEG cortical activity recorded in response to STN stimulation. STN low-frequency stimulation (LFS) at 3 and 5 Hz enables the detection of cortical responses in 333 and 200 ms windows. The EEG recordings were separated into trials defined as time intervals of 50 ms before to 150 ms after the onset of STN stimulus; 2,400 trials were averaged for each contact (Figures 1C–F). The ERP was defined as the average of trials per contact.

The ERPs of all the patients were averaged, and standard error was calculated per channel and per region of interest (ROI) for each area of stimulation. A topoplot was used to visualize differences in average brain activity over the scalp over time per stimulation area.

Each ERP was divided into four time windows, predefined according to a visual inspection of the raw data: very early components (5–25 ms), early components (45–55 ms), middle components (50–100 ms), and late components (100–149 ms).

The ERPs of 64 channels were divided into nine ROIs: left frontal, medial frontal, right frontal, left fronto-central, medial fronto-central, right fronto-central, left occipital parietal, medial occipital parietal, and right occipital parietal. A *z*-score on the ERP was calculated for each patient and each of the 64 channels for all conditions.

Engineered features were calculated for each time window (*n* = 4) per subject in both the individual channels (*n* = 64) and the average of each ROI (*n* = 9). The engineered features calculated were: max and min peak amplitude, max and min peak latency, peak to peak (max peak–min peak), area under the curve (AUC), and energy (squared of the defined window integral).

### Single-Feature Analysis

A single-feature analysis was performed to select the optimal model that differentiates between stimulation location based on the ERP extracted engineered features. The differentiation between areas of stimulation was both within the STN (that is, differentiating between the DLR and the VMR), and in and outside the STN (that is, differentiating between the DLR and ZI or the VMR and ZI). An independent *t*-test was conducted to compare differences in the value of each engineered feature.

The single engineered features were evaluated by 5-fold cross-validation (CV). In the 5-fold CV, we generated a receiver operator characteristic (ROC) curve model. We calculated the cut-off for each feature value and measured whether it could predict the location of the contact, given a two-location classification (ZI vs. DLR, ZI vs.VMR, and DLR vs.VMR).

### Machine Learning Analysis

We used ML classification models to differentiate between stimulation locations based on the engineered features. To avoid overfitting these models, we reduced the number of features as an input, examining the best 5, 10, 15, 20,25, 30, 40, 60, and 80 engineered features that both displayed the greatest dependence on the target class (ZI vs. DLR vs. VMR location) and that were distinct from one another. These features were selected by the Minimal Redundancy, Maximal Relevance (MRMR) feature selection algorithm (Peng et al., 2005).

For two-class and multiclass analyses, we used the SVM algorithm with a linear kernel. In the former, the SVM was used to differentiate between the binary classification both within the STN (between the DLR and the VMR) and in and outside the STN (between the DLR and ZI or the VMR and ZI). In the latter, we used two types of SVM model (One vs. Restand One vs. One) to distinguish all three stimulation locations (ZI vs. DLR vs.VMR). We examined the C parameters of SVM (a regularization parameter) as one of six values, 0.001, 0.01, 0.1., 0.5, 1, or 2, and performed 5-fold cross-validation (CV) to evaluate the models. In each iteration, the trained data were 4- out of 5-fold, leaving 1-fold for validation. A different fold was chosen for this validation in each iteration, with the CV result being the average of these five iterations. This technique was used to find the optimal linear hyperplane classifier to separate between the defined classes with maximal margin.

All EEG processing procedures, including preprocessing, engineered features extraction, and MRMR feature selection algorithm, were performed with Matlab (version 2016b; MathWorks, Natick, MA, United States). The remaining procedures used Python 3.6, NumPy, Seaborn, Statsmodels, Matplotlib, and Skclearn libraries.

De-identified data and relevant analysis code will be shared with other research groups, upon request, for a collaborative study.

## Results

### STN ERP Pattern Is Altered by Stimulation Location in ZI, DLR, or VMR

**Figure 2:**
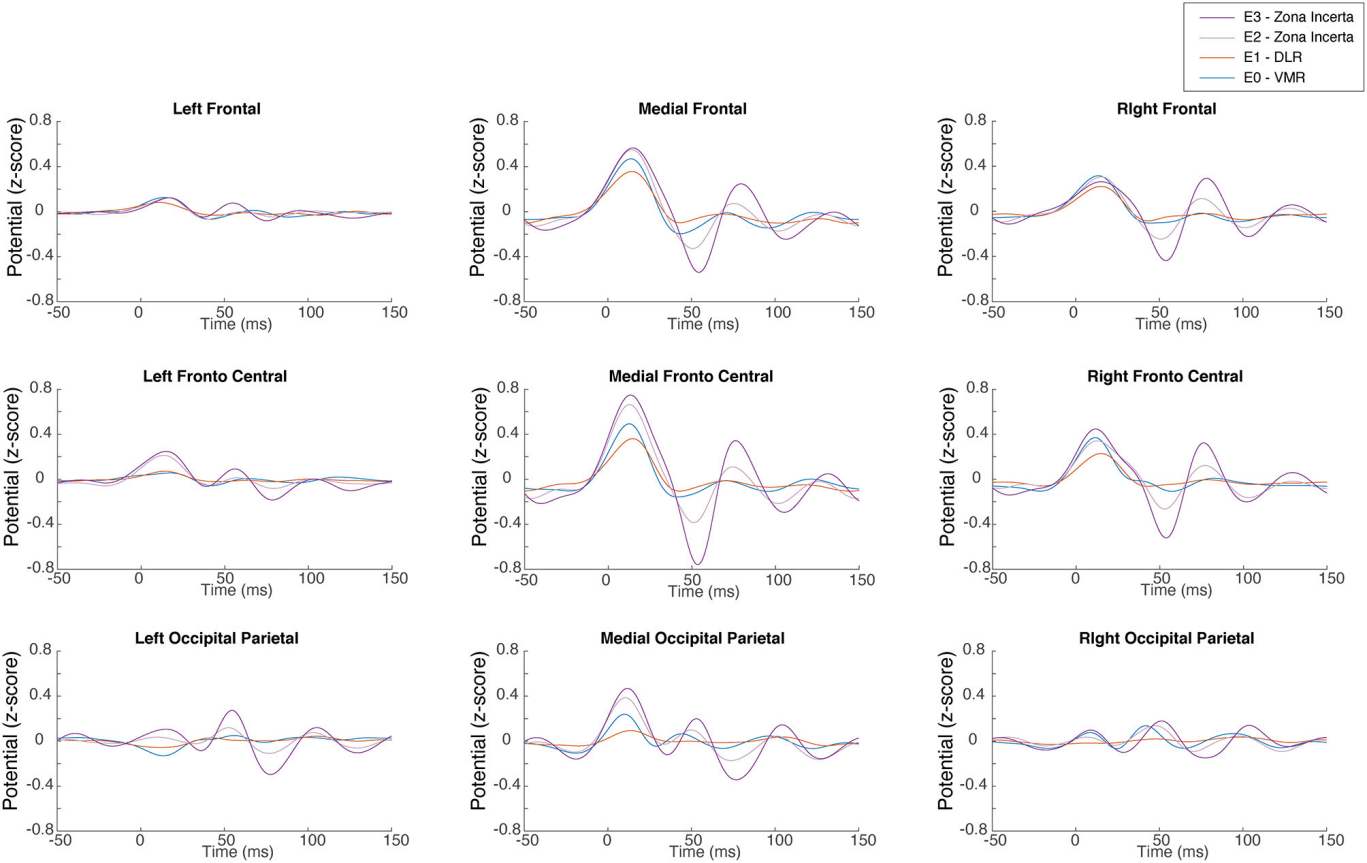
Subthalamic nucleus (STN) evoked response. A representative single subject demonstrates the EEG evoked response in each of the nine regions of interest to low frequency right STN stimulation for each of the four lead contacts. It can also be observed that the signal arises slightly before stimulus onset. Rather than a detection error, this results from the effect of the filter on the interpolated signal, which replaced the stimulus artifact. DLR, dorso-lateral region; VMR, ventro-medial region.

The stimulation of each DBS contact located in a specific area inside and outside the STN (ZI, DLR, and VMR) produces a unique STN ERP pattern. Figure 2 shows an example of the STN ERP recordings of one patient in nine ROIs with DBS stimulation of ZI, DLR, and VMR. Although the ERP pattern is generally similar to that of ZI, DLR, and VMR stimulation, the amplitudes and peak times differ for each area of stimulation. In this example, the highest energy was recorded in the medial-fronto-central area.

**Figure 3:**
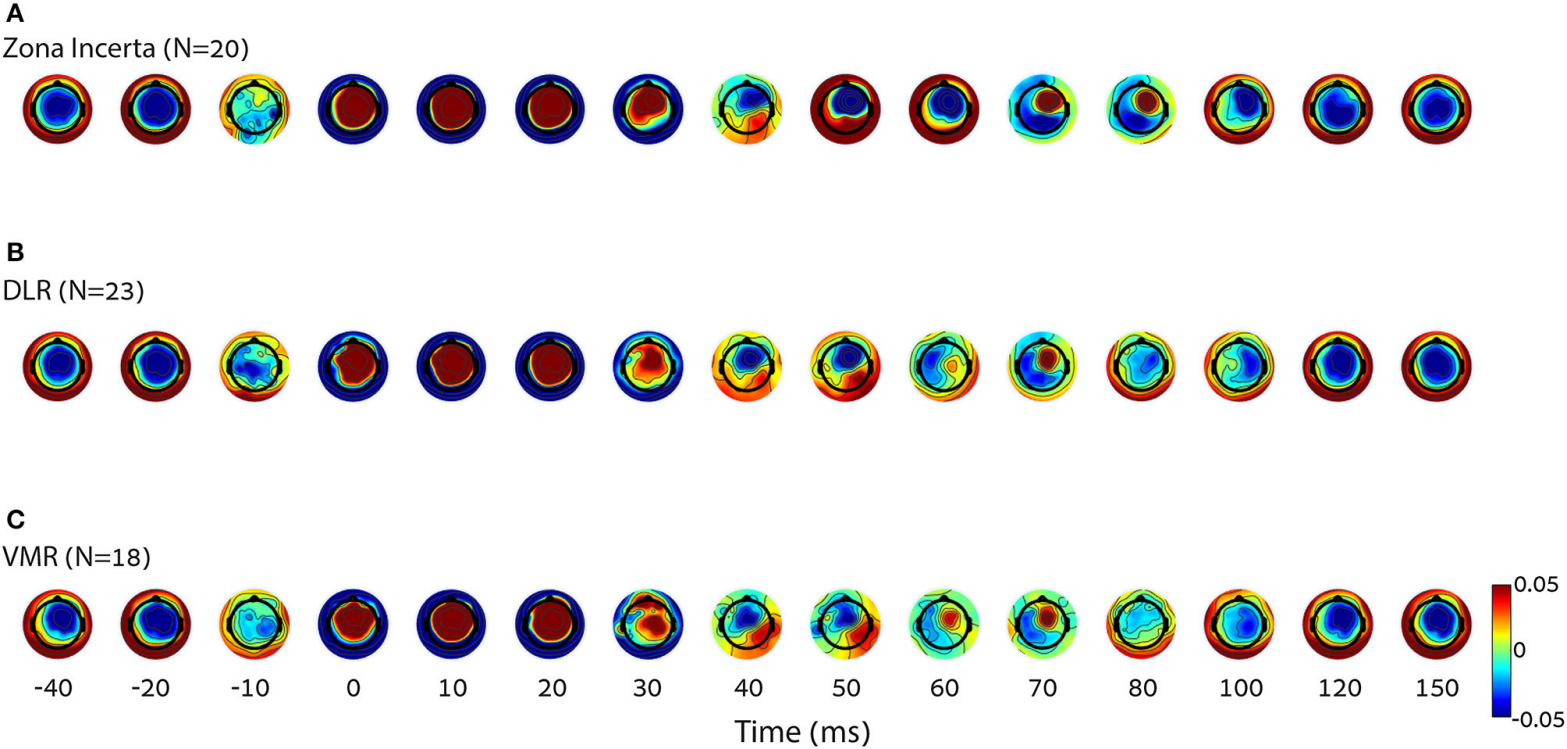
Differences in cortical activity per condition. The average of group topoplot 40 ms before and 150 ms after stimulation reveals a different cortical activity in terms of spatial and time specifics, mostly around 50–100 ms, elicited by DBS in three right STN locations [top down: **(A)** zona incerta (*N* = 20), **(B)** DLR (*N* = 23), **(C)** VMR (*N* = 18)]. The X axis is nonlinear to emphasize the relevant parts. It can also be observed that the signal arises slightly before stimulus onset. Rather than a detection error, this results from the effect of the filter on the interpolated signal, which replaced the stimulus artifact. DLR, dorso-lateral region; VMR, ventro-medial region.

Figure 3 shows topographic plots (topoplots) of the average amplitude of all the subjects over time (from 40 to 150 ms post stimulation). Changes in the time and location domains can be observed both within and without the STN (Figure 3A vs. Figure 3B or Figure 3C), as well as between the DLR and VMR (Figure 3B vs. Figure 3C).

### Single-Feature Analysis of STN ERP Differentiates Inside Vs. Outside STN and Within STN Stimulation Location

**Figure 4:**
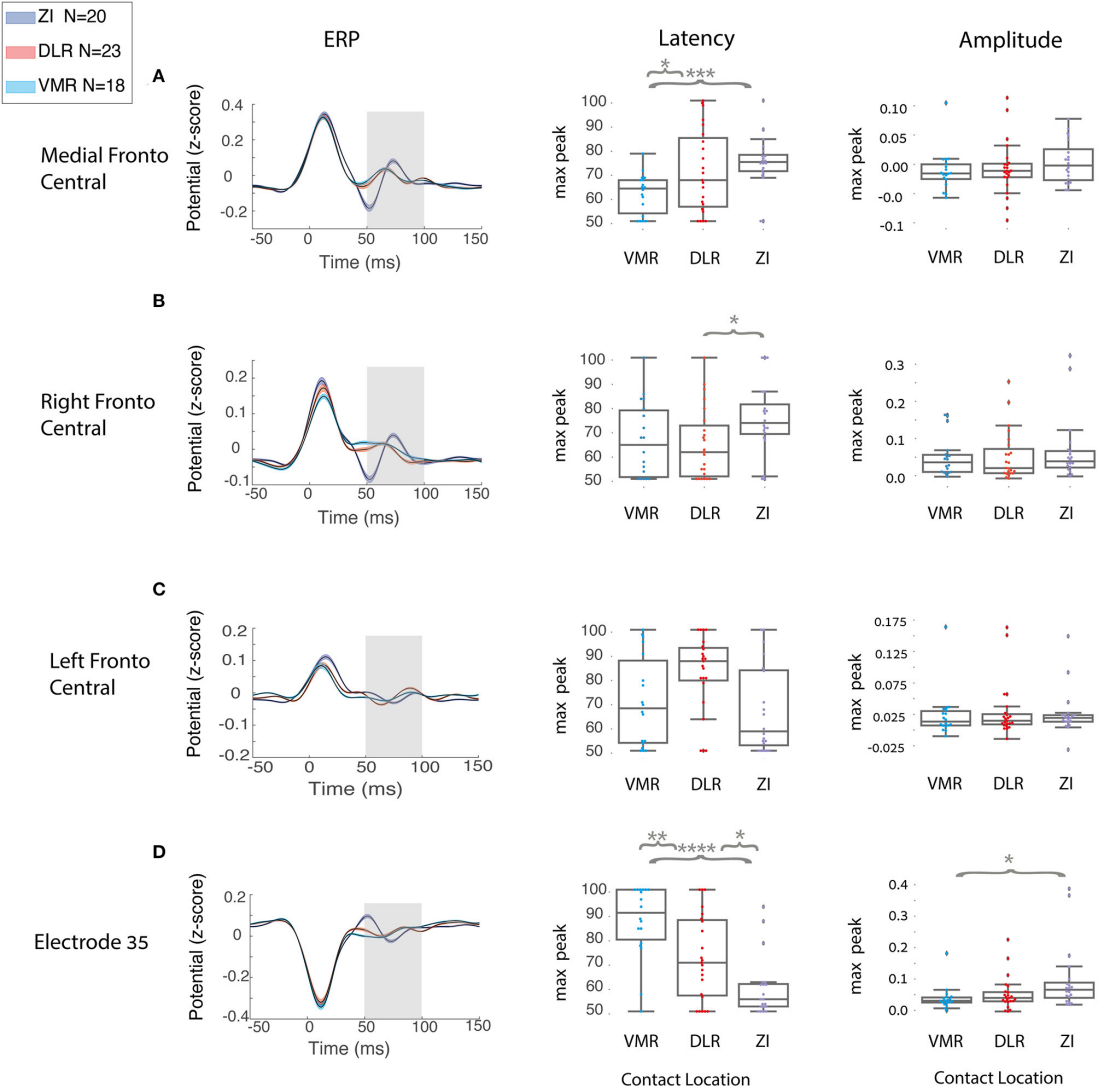
Single feature distinguishes between two STN locations in all the patients. A group analysis of region of interest (ROI) and single electrode in different scalp areas: **(A)** medial fronto-central, **(B)** right fronto-central, **(C)** left fronto-central, **(D)** electrode Oz (at the occipital lobe) reveals a different cortical activity elicited by the DBS in the three STN locations (zona incerta *N* = 20, DLR, *N* = 23, VMR, *N* = 18) represented by the three colors, purple, red, and light blue, respectively. Left column:group ERP average (black line) with standard error (SE) for each STN location; gray rectangle represents the time of interest. Middle and right columns: respectively, group distribution of latency and amplitude at the time of interest. Each distribution is plotted with box and whisker, the ends of each box are the upper and lower quartiles, with the box spanning the interquartile range. The median is marked by a vertical line inside the box, and the whiskers are the two lines outside the box that extend to the highest and lowest observations. Independent *t*-test compared the differences between each two areas. Y-axis is not the same for each row because of differences in amplitude peak between the scalp areas. However, the three STN locations (blue, red, and purple) in each feature are on the same scale, and they are the relevant comparison. Independent *t*-test compared the differences between each two areas (*p < 0.05, **p < 0.01, ***p < 0.001, ****p < 0.0001).

A single-feature analysis of STN ERP, selecting one ROI or one electrode, specific time window and electrophysiological feature, can potentially differentiate areas of STN stimulation and reveal which electrophysiological features influence the ML model most. Figure 4 and Table 2 show examples of single-feature analysis in four recording areas: three ROIs and one single electrode. For each recording area, one time of interest for analysis (gray shadows in left column) and two features (latency and amplitude of the maximal or minimal peak) were selected. These show that single-feature analysis of STN ERP can differentiate between areas of stimulation both within the STN (DLR vs. VMR) and inside and outside the STN (DLR vs. ZI or VMR vs. ZI). The medial fronto-central area in the 50–100 ms post stimulus time window, for example, reveals a significant change in latency between ZI and VMR (with a median latency of 77 and 67 ms, respectively, *t*-value = −4, *p* < 0.0005, see Figure 4A). An ROC curve prediction analysis shows the differentiation between the ZI and VMR, with AUC of 0.86 for the training set and 0.85 for the CV test set (Table 2, column I). Similarly, the right fronto-central area (ipsilateral to stimulation) in the 50–100 ms post stimulus time window reveals a significant change in latency between ZI and DLR (with a median latency of 73.5 and 62 ms, respectively; *t*-value = −2.3; *p* < 0.02; see Figure 4B). An ROC curve prediction analysis demonstrates the differentiation between the ZI and DLR, with AUC of 0.69 for the training set and 0.65 for the CV test set (Table 2, column II). Interestingly, not only ROIs but also single electrodes can differentiate stimulation location. Examining electrode-Oz (occipital lobe) between 50 and 100 ms of post stimulus time window, for example, reveals a significant change in latency between DLR and VMR (median latency of 71 and 92 ms, respectively; *t*-value = 2.9; *p* < 0.01; see Figure 4D). An ROC curve prediction analysis shows the differentiation between the DLR and VMR, with AUC of 0.75 for the training set and 0.73 for the CV test set (Table 2, column III).

**Table 2:** Single-feature classification.

**Analysis type**	**ROC curve**		
	**I**	**II**	**III**
STN area	VMR vs. ZI	DLR vs. ZI	DLR vs. VMR
Number of features	1	1	1
Feature name	Latency max peak	Latency max peak	Latency max peak
Feature area	Medial fronto-central	Right fronto-central	E35
Feature time of interest	50–100 ms	50–100 ms	50–100 ms
Precision training set-mean (std)	0.84 (±0.03)	0.78 (±0.02)	0.64 (±0.03)
Recall training set-mean (std)	0.89 (±0.06)	0.68 (±0.06)	0.88 (±0.03)
Accuracy training set-mean (std)	0.87 (±0.04)	0.73 (±0.03)	0.73 (±0.02)
AUC training set-mean (std)	0.86 (±0.03)	0.69 (±0.04)	0.75 (±0.02)
Precision CV test set-mean (std)	0.81 (±0.19)	0.71 (±0.2)	0.68 (±0.17)
Recall CV test set-mean (std)	0.90 (±0.2)	0.61 (±0.3)	0.81 (±0.2)
Accuracy CV test set-mean (std)	0.84 (±0.18)	0.65 (±0.1)	0.73 (±0.1)
AUC CV test set-mean (std)	0.85 (±0.18)	0.65 (±0.1)	0.73 (±0.09)

*ROC curve analysis shows one feature can differentiate between two STN areas using training and test sets based on CV 5-folds.*

*CV, cross validation*.

For the AUC ROC curve results described above, recall and precision measurements were calculated for the training and CV test sets (Table 2). It should be noted that single-feature analysis can only use a few EEG electrodes recording (such as the medial fronto-central, right fronto-central, and one electrode in the Oz) to differentiate stimulation location accurately, with most of the single features unable to differentiate stimulation location. No differentiation was found, for example, in the left frontal area (contralateral to stimulation) in any time window or any feature (Figure 4C). In addition, while we used 2,400 trials in this study to improve signal to noise ratio, stability tests show that <500 trials are needed for stable results (Supplementary Figure 3).

### Machine Learning Algorithm Better Differentiates STN Stimulation Location

The SVM algorithm of the STN ERP can better differentiate inside vs. outside and within STN stimulation locations. We used two SVM algorithms: a two-class analysis that differentiates between two regions of stimulation and easily interprets the most important engineered features, and a multiclass analysis that differentiates between all the (three) regions of stimulation. For both, we chose the 25 best engineered features as input.

A two-class linear SVM revealed high classification Inside vs. Outside STN, superior to the single-feature analysis values. Both the DLR vs. ZI classification and VMR vs. ZI classification have shown accuracy, precision, and recall probability of 1 for the training set and similar CV test set values, accuracy, precision, and recall of 0.98, 1, and 1, respectively, in the VMR vs. ZI classification, and 0.82, 0.9, and 0.87, respectively, in the DLR vs. ZI classification (Table 3, columns I-II). The classification of subregion within the STN (DLR vs. VMR classification) also yielded better performance than the single-feature analysis, with accuracy, precision, and recall probability of 0.94, 0.95, and 0.88, respectively, for the training set, and 0.77, 0.9, and 0.83, respectively, for the CV test set (Table 3, column III). In the two-class analysis, we calculated the top five most important electrophysiological features, as demonstrated in Supplementary Figure 2.

**Table 3:** Two- and multiclass classifier results.

**Analysis type**	**Two-class analysis**			**Multiclass analysis**	
	**I**	**II**	**III**	**IV**	**V**
STN area	ZI vs. VMR	ZI vs. DLR	DLR vs. VMR	ZI vs. DLR vs. VMR	ZI vs. DLR vs. VMR
Model	Linear SVM	Linear SVM	Linear SVM	Linear SVM One-vs.-Rest	Linear SVM One-vs.-One
Number of features	25	25	25	25	25
C parameter	0.01	0.1	0.01	0.5	0.5
Precision training set	1 (±0.00)	1 (±0.00)	0.95 (±0.06)	1 (±0.00)	1 (±0.00)
Recall training set	1 (±0.00)	1 (±0.00)	0.88 (±0.08)	1 (±0.00)	1 (±0.00)
Accuracy training set	1 (±0.00)	1 (±0.00)	0.94 (±0.05)	1 (±0.00)	1 (±0.00)
Precision CV test set	1 (±0.00)	0.9 (±0.2)	0.9 (±0.1)	0.94 (±0.1)	0.93 (±0.2)
Recall CV test set	1 (±0.00)	0.875 (±0.3)	0.833 (±0.2)	0.91 (±0.1)	0.92 (±0.2)
Accuracy CV test set	0.98 (±0.05)	0.82 (±0.2)	0.77 (±0.2)	0.82 (±0.3)	0.81 (±0.2)

The multiclass analysis yielded high accuracy and very high precision and recall results. We used two SVM multiclass analysis models: One vs. Rest (OvR) and One vs. One (OvO). The SVM OvR analysis showed accuracy, precision, and recall probability of 1 for the training set, and accuracy of 0.82, precision of 0.94, and recall of 0.91 for the CV test set (Table 3, column IV). Similarly, the SVM OvO showed accuracy, precision, and recall of 1 for the training set, and accuracy of 0.81, precision of 0.93, and recall of 0.92 for the CV test set (Table 3, column V).

## Discussion

Our results demonstrate that monopolar STN stimulation evokes a distinct EEG cortical activity that can predict the location of stimulation outside and within the STN. The latency of the DBS evoked response was found to be the best single biomarker for predicting the location of STN stimulation. The medial fronto-central and right-fronto-central areas of the DBS evoked responses were found to be most significant, and most significant for predicting the location of STN stimulation. A novel ML algorithm that used the top 25 engineered features revealed a reliable classification of the ZI, VMR, and DLR areas with precision, recall, and accuracy of 0.94, 0.91, and 0.82, respectively, for the CV test set. These findings imply that an ML model of noninvasive EEG biomarkers can be used to localize DBS contacts in the ZI, VMR, and DLR STN subregions.

### DBS Evoked Responses Can Non-invasively Locate DBS Lead Within the STN

The first level of STN DBS electrode localization is defining the area inside and outside the STN. The second level is defining the STN subregions. The STN can be divided into two subareas, the DLR motor region and the VMR associative-limbic region. The dorso-lateral STN is characterized by a high beta oscillatory activity and is functionally related to the motor cortex and supplementary motor area. The ventro-medial STN is characterized by an alpha-theta oscillatory activity region and a non-beta oscillatory region, and is related to the associative-limbic regions and prefrontal cortex (Zaidel et al., 2010; Horn et al., 2017; Rappel et al., 2020). It is, therefore, important to accurately localize the DBS electrode in the motor area of the STN (DLR) for treatment of movement disorders, and in the associative-limbic area of the STN (VMR) for treatment of emotional and cognitive disorders. Although anatomy-based imaging tools can differentiate the STN inside and outside areas, functional physiology tools are needed to differentiate further the STN subregions. Today, invasive intraoperative physiological microelectrode recordings can accurately localize DBS electrodes within the STN. We argue here that noninvasive physiological EEG ML tools can do so as well.

Ashby et al. (2001) and Baker et al. (2002) were the first to report cortical evoked responses to STN stimulation. Some years later, Mackinnon et al. (2005) showed that the evoked response of the “therapeutic contacts” (first and second DBS contacts) is of lower amplitude than that of the “non-therapeutic contacts” (third and fourth DBS contacts). Eusebio et al. (2009) determined that cortical evoked responses to STN stimulation represent the STN-cortical circuit. They described the cortical evoked activity as consisting of a series of diminishing waves with a natural frequency of around 20 Hz. Miocinovic et al. (2018) have shown a correlation between cortical activity and location within the STN. They recorded cortical STN evoked responses by invasive Electrocorticography (EcoG) array, and demonstrated that ventral STN DBS contacts produce shorter evoked response latencies than dorsal STN DBS contacts. The findings of these studies are in line with our results (Figure 4A). Methodologically, we enlarged our database by recording a relatively long period of time from each contact (8 min), thus, collecting 2,400 trials for each contact and 9,600 trials for each patient. This enabled the improvement of the signal-to-noise ratio and a more accurate study on the cortical activity, suggesting that a non-invasive EEG tool can differentiate subregions within the STN. Stability tests show that <500 trials are needed for stable results, that is, future intraoperative recordings of <2 min (per contact) may be sufficient for our suggested method for noninvasive EEG that can differentiate subregions within the STN.

### Monopolar STN Stimulation Can Produce Distinct Cortical Signals

The majority of rodent and human DBS cortical evoked response studies have performed bipolar stimulation configurations (Ashby et al., 2001; Baker et al., 2002; Mackinnon et al., 2005; Walker et al., 2012; Kelley et al., 2018; Kibleur and David, 2018; Kumaravelu et al., 2018; Miocinovic et al., 2018; Iacono et al., 2019; Romeo et al., 2019). Bipolar stimulation can potentially differentiate between the stimulus artifact and cortical activity (Baker et al., 2002; Walker et al., 2012). Our study performed mono-polar stimulation and found significant cortical evoked responses. It has been recently shown that both monopolar and bipolar STN stimulation can evoke comparable cortical responses (Miocinovic et al., 2018). These cortical responses represent the STN-cortical activity with similar amplitude and latency for the components after the stimulus artifact >1 ms.

We suggest that the later evoked response components (>5 ms) are not affected by the stimulus artifact (<1 ms) in either bi- or monopolar stimulations. In addition, our results demonstrate that the cortical evoked response does not necessarily correlate with the distance between stimulating lead and cortex. As shown in Figure 1C, for example, the most ventral contact, furthest from the cortex (E0), demonstrates a higher cortical activity than the adjacent E1 lead, closer to the cortex. In addition, in the medial fronto-central ROI (Figure 4A), the DLR peak latency is significantly longer than the VMR peak latency (69 and 67 ms, respectively), although DLR is closer to the cortex. Similarly, in the right fronto-central ROI (Figure 4B), the ZI peak latency is significantly longer than the VNR and DLR peak latencies (73.5 vs. 62 and 64, respectively), although the ZI is closer to the cortex. We, therefore, conclude that EEG cortical activity in response to monopolar STN stimulation is not simply a stimulus artifact but represents a genuine cortical activity.

Another advantage of monopolar stimulation is the lower stimulation energy (amplitude) needed to show a cortical evoked response. Previous EEG studies have suggested that the comparison of bi- and mono-polar stimulations requires a 30% increase in bipolar amplitude to achieve similar results (Mackinnon et al., 2005). Similarly, Zumsteg et al. (2006) showed that the cortical response amplitude of thalamic stimulation in patients with epilepsy is four times higher with monopolar than bipolar stimulation. One ECoG study has reported that an amplitude increase of up to 280% is needed for bipolar STN stimulation to achieve the same volume of cortex component activation as monopolar STN stimulation in terms of latency and amplitude (Miocinovic et al., 2018). This study also indicates that the polarity of the bipolar stimulation may affect the cortical components. Opposite polarity bipolar stimulation changed the cortical activity amplitude by more than 25% (Miocinovic et al., 2018). Previous EEG studies performing bipolar stimulation with inadequate stimulation energy may, thus, have missed the cortical activity we report here. Previous EEG studies performing bipolar simulation with inadequate stimulation energy may, thus, have missed the cortical activity we report here.

In our study, -polar stimulation evoked cortical activity with a spatial resolution that supports the known anatomic STN-cortical networks. STN stimulation mainly evokes sensorimotor areas, such as the medial fronto-central and right fronto-central areas, ipsilateral to stimulation (Chen et al., 2020; Gunalan and McIntyre, 2020; Johnson et al., 2020). No muscle contraction was observed in response to stimulation. We did not observe any stimulation effect on speech or gaze either. We can, therefore, rule out any current spread of STN stimulation to surrounding structures.

The origin of STN-evoked responses likely results from both antidromic and orthodromic responses. The short responses (5–10 ms) may indicate an antidromic direct activation of the cortex. The middle and late latency (>10 ms) cortical evoked responses may indicate an orthodromic activation of polysynaptic synapses at the basal ganglia and thalamocortical pathways. It is possible that the differences in evoked responses between ZI, DLR, and VMR reflect a difference in input and output connectivity (Smith et al., 1994; Hashimoto et al., 2003; Miocinovic et al., 2006; Sanders and Jaeger, 2016; Gunalan et al., 2017). Our results reveal that the main differences between locations are seen in the time frame of 50–100 ms and are likely the result of an orthodromic pathway. Similarly, an ECoG study by Miocinovic et al. (2018) showed that a cortical activity 10–100 ms after STN stimulation is related to the orthodromic pathway.

### Toward a Tailored ML Tool for DBS Localization and Programming

Our suggested ML model of noninvasive EEG biomarkers to localize DBS contacts in STN subregions may assist in both intraoperative electrode localization and postoperative contact selection. In our study, we used awake postoperative EEG recordings. Most recently, Irwin et al. (2020) have reported a similar cortical evoked response to STN stimulation in both awake patients and those under general anesthesia. We, therefore, suggest that future studies on our ML model with intraoperative EEG recordings in awake and anesthetized patients will be clinically useful for intraoperative patient-specific electrode localization. Similarly, our ML model can be used postoperatively for contact selection in patients and contribute information to future closed-loop DBS models (Ramirez-Zamora et al., 2018; Vissani et al., 2020; Sand et al., 2021).

As indicated, we performed both single-feature and ML analyses. The single-feature analysis enabled a good classification of two STN subregions, ZI-DLR and ZI-VMR. The top single features found are in line with our current physiologic and anatomic knowledge of the subthalamic-cortical networks. The ZI-DLR and ZI-VMR localization, for example, was based on the latency of the evoked response and on sensorimotor cortical areas. The ML analysis enabled us to localize more accurately all the three tested STN locations (ZI, DLR, and VMR). The advantage of the ML model is finding the best features of the EEG signals and calculating the relative contribution and importance of each to predicting STN localization. It is not surprising that our ML model increased prediction accuracy in our study: representations learned by ML in various areas (computer vision, speech recognition, natural language understanding, and more) as well as EEG analysis in epilepsy are significantly superior to manually tuned features constructed by experts during years of research (Shalev-Shwartz and Ben-David, 2014; Qaraqe et al., 2015; Zhang et al., 2016; Dirodi et al., 2019; Jang and Cho, 2019; Kramer et al., 2019). Our suggested algorithm automatically standardizes the data and minimizes noise artifacts without the need for manually reviewing or visually inspecting the data. Our ML model is, thus, easy to apply clinically, since it requires no special electrophysiological or medical expertise.

It should be noted that, like other ML studies, ours is limited by the size of the database. Although we recruited a relatively large patient sample and recorded it for long periods of time, a larger dataset will yield higher prediction values. We validated our results in more than one medical center, recruiting patients from two hospitals where their DBS procedures were performed by two independent neurosurgery teams, and their STN electrode localization was evaluated by independent electrophysiology experts. Future studies should include more patients and more medical centers.

## Conclusions

We show here that an ML model of noninvasive EEG biomarkers can differentiate STN subregions. DBS contact localization is crucial for the clinical outcome of STN DBS procedure (Israel and Bergman, 2016). In addition, postoperative DBS contact identification is important for postoperative contact selection. Future studies are needed to implement this novel noninvasive tool in the operating room, while patients are awake and while under anesthesia. EEG recordings should also be considered as an important element in future closed-loop DBS tools.

## Data Availability Statement

The raw data supporting the conclusions of this article will be made available by the authors, without undue reservation.

## Ethics Statement

This study was authorized by the local IRB Committee of Hadassah Medical Center [no. 0403-13-HMO, NIH clinical trials registration (no. NCT01590056)] and local IRB Committee at Sheba Medical Center (no. 3496-16-SMC, NIH clinical trials registration NCT01590056). The patients/participants provided their written informed consent to participate in this study.

## Author Contributions

DS: research concept, electrophysiological data recording, data organization, statistical analysis, and writing the manuscript. DA: research concept, patient assessment and monitoring, pre- and postoperative DBS procedures, electrophysiological data recording, and reviewing the manuscript. MA: patient assessment and monitoring, pre- and postoperative DBS procedures, and electrophysiological data recording. OM: electrophysiological intraoperative recording. ZI: the leading neurosurgeon of the research group, performing the surgeries and contributing to the intra-operative electrophysiological recordings at Hadassah Medical Center. SH-B: patient assessment and monitoring and pre- and postoperative DBS procedures. SI-K: patient assessment and monitoring, pre- and postoperative DBS procedures, and reviewing the manuscript. ZP: research concept, electrophysiological data recording, and reviewing the analysis. AG: research concept and reviewing the analysis. HB: the leading electrophysiologist of the research group: contributing to the research concept, electrophysiological data recording, overseeing and reviewing the analysis, and writing the manuscript. RE: leading the project, recruiting patients, and overseeing and reviewing the analysis and writing. DS, HB, and RE take responsibility for the integrity of the data and accuracy of the data analysis. All authors have read and approved the final version of the manuscript.

## Funding

This study was partially supported by grants from the Magnet Program of the Office of the Chief Scientist (OCS) of Israel's Ministry of Economy (to HB and AG) and the Israel Science Foundation-ISF, no. 2128/19 (RE).

## Conflict of Interest

DS is an employee of Elminda Ltd. ZP and AG are consultants of and have financial interest in Elminda Ltd. HB is a consultant of AlphaOmega. At the time of the study experiments, SI-K worked at the Sheba Medical Center and, since November 2020, has been an employee of NeuroDerm. The remaining authors declare that the research was conducted in the absence of any commercial or financial relationships that could be construed as a potential conflict of interest.

## Publisher's Note

All claims expressed in this article are solely those of the authors and do not necessarily represent those of their affiliated organizations, or those of the publisher, the editors and the reviewers. Any product that may be evaluated in this article, or claim that may be made by its manufacturer, is not guaranteed or endorsed by the publisher.
