# Supplementary material for: Deep Brain Stimulation Can Differentiate Subregions of the Human Subthalamic Nucleus Area by EEG Biomarkers
Source: Front Syst Neurosci. 2021 Oct 20;15:747681. doi: 10.3389/fnsys.2021.747681 (PMC8565520; doi:10.3389/fnsys.2021.747681)
Supplement: Supplementary file 1 [file Data_Sheet_1.pdf]

Supplementary Material

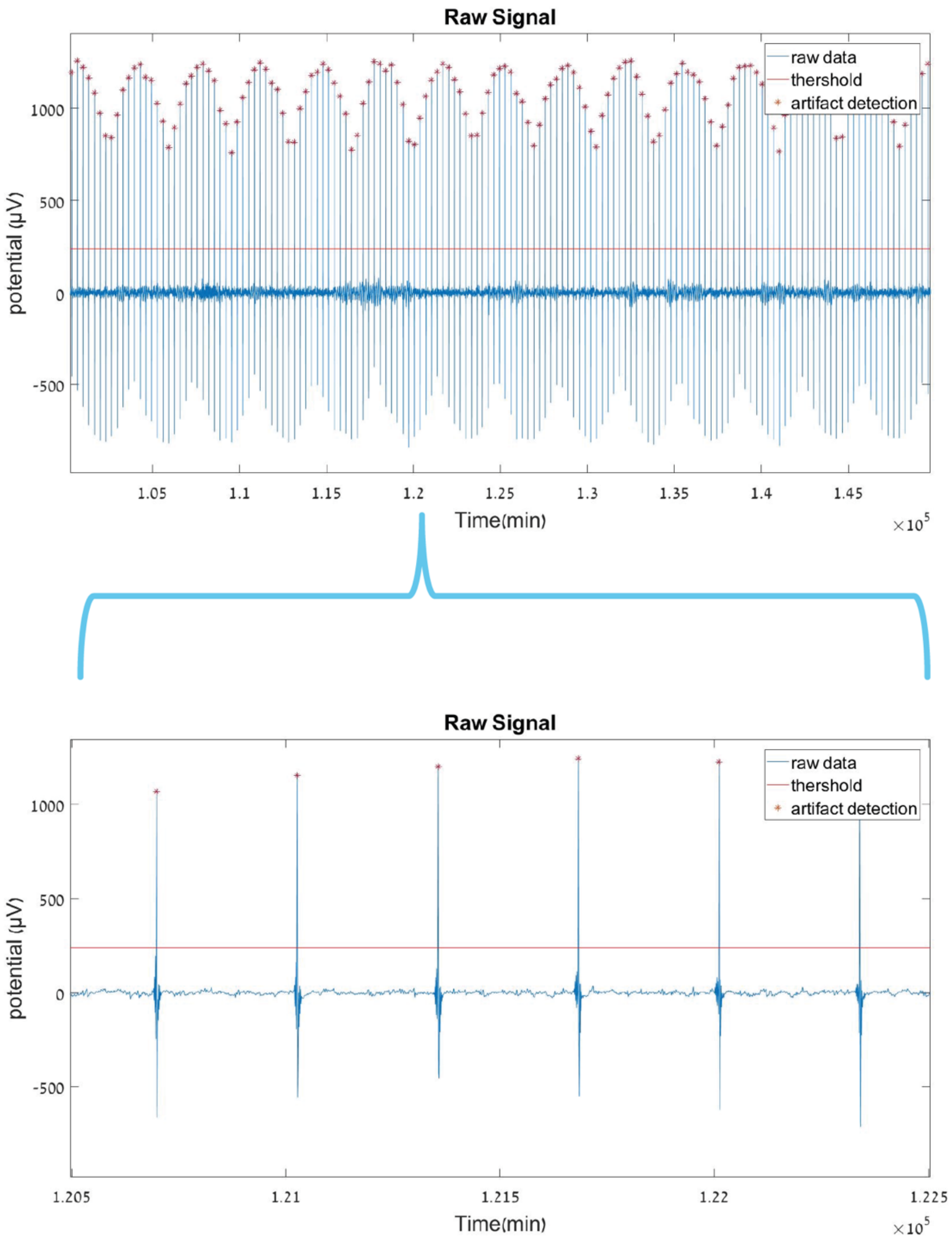

Figure S1: Artifact Detection

A representative subject (S6) demonstrates the raw EEG with DBS stimulus artifact. The threshold for artifact detection was defined above 3* standard deviations (SD) of the raw signal.

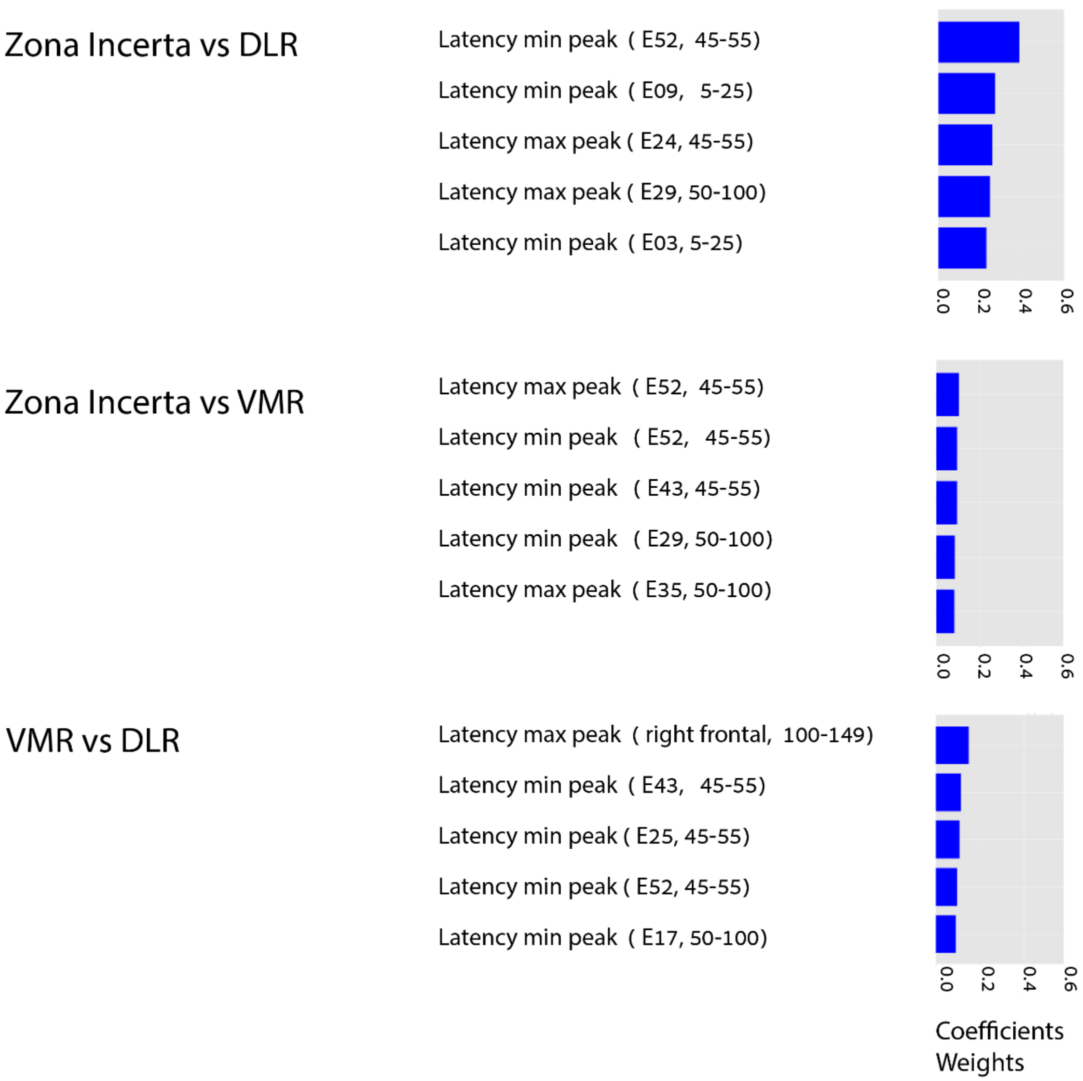

Figure S2: SVM Model Using ERP Biomarkers Differentiates Both Inside vs. Outside STN and Subregions within the STN

The top five features selected by MRMR, used as input for each SVM model, reveal that each region has its own biomarkers to discriminate between the areas. Each feature name integrates the engineered feature, electrode and time of interest in ms.

The X-axis is the actual coefficient weights assigned to the linear SVM model.

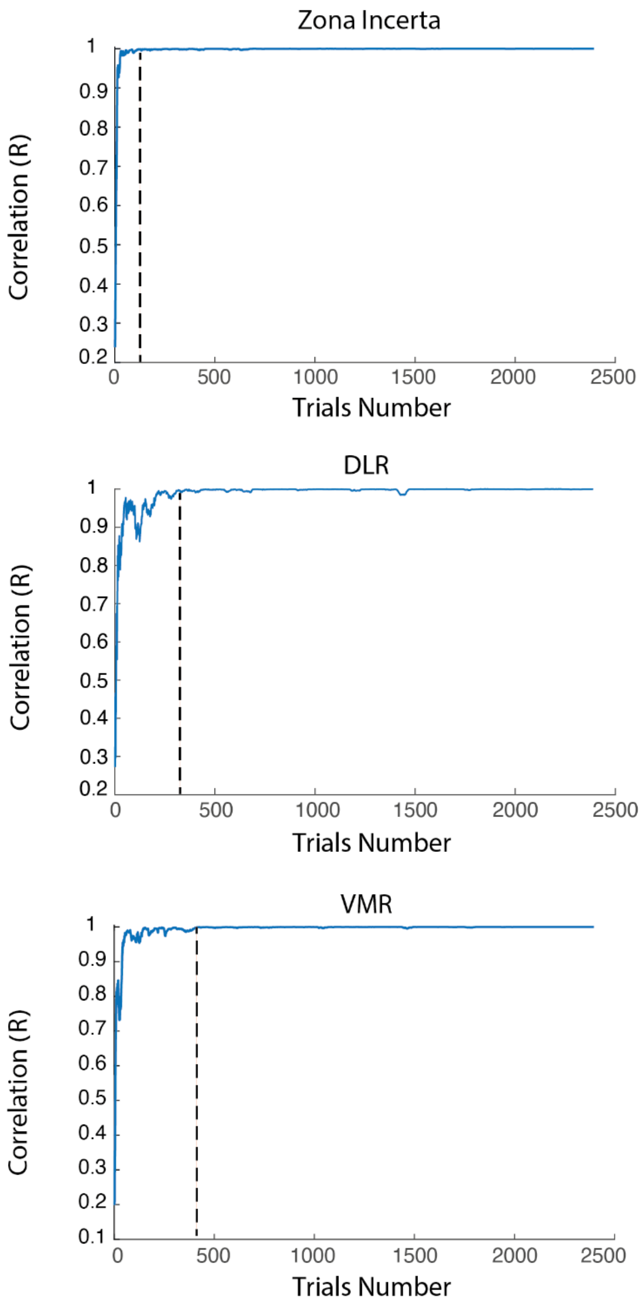

Figure S3: Only a Few Hundred Trials Signals are Needed for a Stable ERP Signal

The correlation coefficient between the ERP averaged by X trials vs. the ERP averaged by X-50 trials shows stability effect after ~200 trials for the Zona Incerta (top), after ~300 trials for the DLR (middle), and after ~500 trials for the VMR (bottom) in a representative subject (no S8) in the middle fronto-central, displayed by the blue line. Marker showing the correlation tends to be 1 until the end of the trials, displayed by the black dashed line.
